# miR-18a reactivates the Epstein-Barr virus through defective DNA damage response and promotes genomic instability in EBV-associated lymphomas

**DOI:** 10.1186/s12885-018-5205-9

**Published:** 2018-12-29

**Authors:** Pengfei Cao, Meili Zhang, Lujuan Wang, Buqing Sai, Jiuqi Tang, Zhaohui Luo, Cijun Shuai, Liyang Zhang, Zheng Li, Yanjin Wang, Guiyuan Li, Juanjuan Xiang

**Affiliations:** 1 Key Laboratory of Carcinogenesis of Ministry of Health, Xiangya Hospital, Central South University, Changsha, 410078 Hunan China; 2 Key Laboratory of Carcinogenesis and Cancer Invasion of Ministry of Education, Cancer Research Institute, Central South University, Changsha, 410078 Hunan China; 3 Hunan Key Laboratory of Nonresolving inflammation and Cancer, Desease Genome Research Center, The Third Xiangya Hospital, Central South University, Changsha, 410013 Hunan China; 4 State Key Laboratory of High Performance Complex Manufacturing, Central South University, Changsha, 410083 Hunan China; 5 People’s Hospital of Dezhou, Dezhou, 253045 Shandong China; 6 Department of Neurosurgery, Xiangya Hospital, Central South University, Changsha, 410078 Hunan China

**Keywords:** miR-18a, EBV reactivation, DNA damage response, Genomic instability

## Abstract

**Background:**

The Epstein-Barr virus (EBV) is closely associated with several types of malignancies. EBV is normally present in the latent state in the peripheral blood B cell compartment. The EBV latent-to-lytic switch is required for virus spread and virus-induced carinogenesis. Immunosuppression or DNA damage can induce the reactivation of EBV replication. EBV alone is rarely sufficient to cause cancer. In this study, we investigated the roles of host microRNAs and environmental factors, such as DNA-damage agents, in EBV reactivation and its association with lymphomagenesis.

**Methods:**

We first analyzed the publicly available microRNA array data containing 45 diffuse large B-cell lymphoma patients and 10 control lymph nodes or B cells with or without EBV infection. In situ hybridization for miR-18a and immunohistochemitry were performed to evaluate the correlation between the expression of miR-18a and nuclear EBV protein EBNA1 in lymphoid neoplasm. The proliferative effects of miR-18a were investigated in EBV-positive or –negative lymphoid neoplasm cell lines. EBV viral load was measured by a quantitative real-time EBV PCR and FISH assay. The genomic instability was evaluated by CGH-array.

**Results:**

In this study, we analyzed the publicly available microRNA array data and observed that the expression of the miR-17-92 cluster was associated with EBV status. In situ hybridization for miR-18a, which is a member of the miR-17-92 cluster, showed a significant upregulation in lymphoma samples. miR-18a, which shares the homolog sequence with EBV-encoded BART-5, promoted the proliferation of lymphoma cells in an EBV status-dependent manner. The DNA-damaging agent UV or hypoxia stress induced EBV activation, and miR-18a contributed to DNA damaging-induced EBV reactivation. In contrast to the promoting effect of ATM on the lytic EBV reactivation in normoxia, ATM inhibited lytic EBV gene expression and decreased the EBV viral load in the prescence of hypoxia-induced DNA damage. miR-18a reactivated EBV through inhibiting the ATM-mediated DNA damage response (DDR) and caused genomic instability.

**Conclusions:**

Taken together, these results indicate that DNA-damaging agents and host microRNAs play roles in EBV reactivation. Our study supported the interplay between host cell DDR, environmental genotoxic stress and EBV.

**Electronic supplementary material:**

The online version of this article (10.1186/s12885-018-5205-9) contains supplementary material, which is available to authorized users.

## Background

The Epstein-Barr virus (EBV) infects nearly all humans. Although EBV injection is asymptomatic in most individuals, EBV is closely associated with several types of malignancies, including lymphomas, nasopharyngeal carcinoma (NPC), gastric adenocarcinoma and gastric lymphoepithelioma-like carcinoma [1–3]. Due to the tropism for B lymphocytes, the most common forms of EBV-associated lymphoproliferative disorders are B-cell lymphomas: Hodgkin lymphomas (HL), non-Hodgkin lymphomas (NHLs), including Burkitt lymphoma (BL) and diffuse large B-cell lymphoma (DLBCL) [4]. EBV also plays a complex and multifaceted role in T/NK cell lymphomas [5].

The virus is present in two stages: an active lytic state and latent state, and the virus can only be spread during the active state [6]. During primary EBV infection, a portion of individuals, particularly adolescents, develop infectious mononucleosis (IM). EBV infection in vitro can transform resting B cells into immortalized lymphoblastoid cell lines (LCL).Under normal conditions, EBV is present in the latent state in the peripheral blood B cell compartment. EBV can be reactivated under certain circumstances. Reactivation of the viral replication plays an important role in the development of EBV-associated malignancy. Oral hairy leukoplakia is unequivocally due to lytic EBV infection [7]. Except for hematological malignancies, the lytic form of EBV infection was also observed in malignant breast epithelial cells in certain cases of breast cancer [8]. EBV reactivation-induced cell proliferation and migration caused the relapse of NPC [9]. Elevated levels of EBV DNA is observed in EBV-positive lymphoma patients with active disease but not in EBV-positive patients in remission or those with EBV-negative tumors, indicating that the EBV viral load at lymphoma diagnosis is an indicator of disease activity and biological characteristics associated with a negative prognosis [10].

Environmental factors serve to promote EBV-driven tumors, which are primarily of B cell origin but also of epithelial and NK or T cell origin. Immunosuppression or DNA-damaging agents, including chemotherapy, certain HDAC inhibitors, and radiation, can induce the reactivation of EBV replication [11]. EBV infection in turn has been implicated in DNA damage. Phosphorylated H2AX, a reporter of DNA damage was increased in EBV-carrying cells in the absence of exogenous stimuli [12].

Deregulation of the cellular Myc proto-oncogene is the hallmark of all types of Burkitt lymphoma. The Myc-mediated oncogenic signaling pathway regulates miRNAs, especially miR-17-92 clusters. miR-17-92 is transcribed as a polycistron, which is subsequently processed into 7 mature miRNAs: miR-17-3p and -5p, miR-18a, miR-19a, miR-19b, miR-20a and miR-92a. The miR-17-92 cluster is frequently amplified or overexpressed in lymphoma [13]. Compared with the other miR-17-92 members,which showed distinct functional interplays with Myc, the role of miR-18a remains unexplained. In our previous study, miR-18a was shown to promote malignant progression of nasopharyngeal carcinoma, which is closely related to EBV infection [14]. miR-18a shares a homologous sequence with EBV-encoded BART 5. In this study, we aimed to elucidate the roles of miR-18a in the tumorigenesis of lymphoma and determine whether they are mediated by EBV.

## Methods

### Cell culture and patients samples

Human Burkitt lymphomas cell lines P3HR-1, Raji(EBV positive)and BJAB(EBV negative) were cultured in RPMI-1640 (HyClone, Life Sciences, Logan, UT, USA) supplemented with penicillin G (100 U/mL), streptomycin (100 mg/mL) and 10% fetal calf serum. Cells were grown at 37 °C in a humidified atmosphere of 5% CO_2_ and were routinely sub-cultured. Cells were obtained from Cell Bank of Cancer Research Institute, Central South University and authenticated by STR profiling. Patients diagnosed with lymphoma (*n* = 100) were included in this study. Twenty non-cancerous individuals were enrolled as a control group. All cases enrolled in this study were identified at Xiangya hospital, Central South University, China. The clinical and laboratory characteristics of the cases are summarized in Table 1. The patients were informed of the sample collection and signed informed consent forms. The collection and use of samples were approved by the ethical review committees of Xiangya Hospital, Central South University.

**Table 1:** The clinical, laboratory characteristics of paitents

Clinical and pathological features	n	miR-18a + high	miR-18a -low	*P* Value
Gender				
Men	65	35	30	
female	35	16	19	0.5304
Age				
≥50	55	28	27	
< 50	45	23	22	1.000
Extranodal lymph node metastasis				
Yes	71	43	28	
NO	29	8	21	0.0039
Clinical stage				
I-II	37	12	25	
III-IV	63	39	20	0.0017
EBV(EBNA-1)				
+	51	42	9	
–	49	9	40	< 0.0001

### Analysis of public datasets

The microRNA array datasets (GSE42906, GSE36926) were collected from the National Center for Biotechnology Information’s Gene Expression Omnibus (GEO, NCBI). Using GEO2R of PubMed (http://www.ncbi.nlm.nih.gov/geo/geo2r/), we analyzed changes in the miRNA expression spectrum. Expression of the miR-17-92 cluster and EBV-encoded microRNA was analyzed and visualized by Multiple Experiment Viewer (MeV).

### Immunohistochemistry and in situ hybridization

Lymphomas samples and normal lymph node tissues were fixed and embedded in paraffin wax. Next, 4- to 6-μm thick paraffin sections were defaced followed by hydration. The detailed procedures for the immunohistochemistry for EBNA1 and in situ hybridization for miR-18a have been described in our previous publication [14]. Tissue sections were incubated with primary antibody EBNA1 (Novus Biologicals, Littleton, Colorado, USA) at 4 °C overnight in a humidified chamber. Finally, after dehydration and mounting, the sections were observed and imaged under a microscope (OLYMPUS BX-51, Japan). Goat serum and PBS were used instead of the first antibody as a negative control and blank control respectively. A semi-quantitative scoring criterion for IHC was used in which both the staining intensity and positive areas were recorded.

### Cell cycle analysis

Cells were fixed with 70% ethanol overnight and were incubated in PBS containing 0.5 mg/ml RNase A (Takara, Japan) at 37 °C for 1 h. Cells were treated with propidium iodide at the final concentration of 5 μg/ml (Beyotime Biotechnology, Hangzhou, China) and subjected to flow cytometry analysis.

### Quantitative real-time PCR

Total RNA was isolated from cells using Trizol® reagent (Invitrogen, Carlsbad, CA, USA). cDNA was synthesized from total RNA using the RevertAid First Strand cDNA Synthesis Kit (Fermentas, Waltham, MA, USA). To measure the expression of miR-18a, the microRNA qPCR Quantitation kit was purchased from Takara, Japan. Real-time PCR was performed using the Bio-Rad IQ™^5^ Multicolor Real-Time PCR detection System (Bio-Rad, Berkeley, CA, USA). The data were analyzed using iQ5 software. Relative EBV-related gene expression was determined and normalized to the expression of GAPDH or β-actin (for hypoxic conditions) using the 2^ΔΔCt^ method. miR-18a expression was calculated and normalized to U6 using the 2^ΔΔct^ method. The data are representative of the means of three experiments. Student’s t-test was applied to compare two or more values; *p* < 0.05 indicated that there was a significant difference. The qPCR protocol was as follows: 95 °C for 30 s and 40 cycles of 95 °C for 5 s and 60 °C for 30 s. A final melting curve analysis (60–95 °C) was conducted. The RT-PCR primers are listed below.

ERER1/2 forward:5’-GAGGTTTTGCTAGGGAGGAGA-3′.

ERER1/2 reverse:5’-CACCACCCGGGACTTGTA-3’.

EBNA1 forward: 5’-GAGAAGGGAGACACATCTGGA-3’.

EBNA1 reverse: 5’-CATGGTTATCACCCCCTCTTC-3’.

LMP1 forward:5’-CCCACTCTGCTCTCAAAACC-3’.

LMP1 reverse:5’-GTCCTGTGGGCCATTGTC-3’.

BZLF1 forward:5’-AGCTTATGCACCTCAGC-3’.

BZLF1 reverse:5’-CCGGCTTGGTTAGTCTGTTG-3’.

BMRF1 forward:5’-GCGAGGAAAAGGACATCGT-3’.

BMRF1 reverse:5’-CTTCACTTTCTTGGGGTGCT-3’.

BRLF1 forward:5’-GCTCAGGTCCATCTGTCCAC-3’.

BRLF1 reverse:5’-GGGAGATGGCTGACACTGTT-3’.

GAPDH forward:5’-AGCCACATCGCTCAGACAC-3’.

GAPDH reverse:5’-GCCCAATACGACCAAATCC-3′.

β-actin forward: 5’-ACTGGAACGGTGAAGGTGAC-3′.

β-actin forward: 5’-AGAGAAGTGGGGTGGCTTTT-3′.

Quantitative real-time EBV PCR was performed in samples collected from the media of cells using the Epstein-Barr virus DNA Quantitative Fluorescence Diagnostic Kit (Sansure Biotech, Hunan, China). Viral DNA was extracted, and PCR was performed according to the manufacturer’s instructions. The qPCR protocol was as follows: 94 °C for 5 min, followed by 45 cycles of 94 °C for 15 s and 57 °C for 30 s. The EBV copy number was calculated according to the standard curve.

### EBV purification and fluorescence in situ hybridization

An EBV-specific probe was constructed from EBV and obtained from the productive EBV B-cell lineage P3HR-1, which has been describe in detail previously [15, 16]. Briefly, the culture media were collected from P3HR-1, followed by repeated freezing and thawing for several times. After centrifuged for 20 min at 3000 RPM, the supernatant was collected and filtered with a 0.45-μm membrane filter. EBV viral DNA was extracted using the Qiagen QIAamp Virus MinElute Spin Kit (Qiagen, Valencia, CA, USA). PCR was performed to amplify the 3267 bp of EBV DNA probes. The DNA probes were labeled with biotin-dUTP by the random-primed labeling method (Roche, Mannheim, Germany). The slides were incubated with hybridization mixture containing 50 ng of probe and 5 μg of salmon sperm DNA overnight at 37 °C for 14 h. Following hybridization, the slides were incubated with avidin-FITC for 30 min at room temperature followed by incubation with anti-FITC. After washing, drying and mounting, the slides were examined under fluorescence microscopy (Olympus FSX100).

### Western blotting

The procedures for western blotting have been described in our previous publication [14]. Briefly, approximately 50 μg of protein were separated on SDS-PAGE and was transferred to PVDF membranes (Millipore, MA, USA). The membranes were incubated with the primary antibody overnight at 4 °C followed by a brief wash with PBST and incubation with secondary antibody for 1 h at 37 °C. An anti-GAPDH antibody control was purchased from Millipore (Billerica, MA, USA) and was used as a loading control. Finally, ECL solution (Millipore, MA, USA) was added to cover the blot surface, the signals were captured, and the intensity of the bands was quantified using the Bio-Rad ChemiDoc XRS+ system (Bio-Rad, CA, USA). The antibodies were used as follows: ATM, EBNA1 (Novus Biologicals, USA), γ-H2AX(Abcam, USA), β-actin (Santa Cruz Biotechnology, CA, USA) and GAPDH (cell signaling, USA).

### In vitro cell proliferation assessment

The proliferation of lymphoma cells in suspension was measured using the CCK-8 assay (Beyotime Biotechnology, Hangzhou, China). The cell suspension was inoculated in a 96-well plate. After treatment, 10 μl of CCK-8 solution was added to each well and the plate was incubated for an additional 4 h. Next, the absorbance measured at 450 nm using a microplate reader. The experiment was repeated three times, and six parallel samples were measured each time.

### Hypoxia and UV treatment

The cells were incubated in the hypoxic chamber with 0.1% oxygen, 95% N_2_ and 5% CO_2_ (Don Whitley Scientific, H35 hypoxystation) for 48–72 h. For UV treatment, cells were subjected to UV light (254 nm) for 10 min to cause DNA damages.

### Immunofluorescence analysis

Cells were collected, washed and subsequently fixed in fixation buffer (4% paraformaldehyde in PBS). Next, the cells were permeabilized for 30 min with 0.25% Triton X-100 and blocked for 60 min with 5% BSA. Cells were incubated with anti-γ-H2AX antibody at a 1:800 dilution at 4 °C overnight, washed in TBST 3 times, and incubated with the secondary antibody FITC-conjugated IgG (BD biosciences, USA) at a dilution of 1:300 for 1 h. Next, cells were washed in TBST at room temperature, dried and mounted by mounting medium including the nucleus stain DAPI (Vector Laboratories). The cells were examined by fluorescence microscopy (Olympus FSX100).

### Luciferase assay

Reporter constructs were generated in which the 3’UTR of ATM, wild-type or miR-18a binding-site mutant was cloned downstream of the luciferase open reading frame. Luciferase activity was measured using the Dual-Glo luciferase assay system (Promega). Renilla luciferase activity was normalized to the corresponding firefly luciferase activity. 293 cells were co-transfected with luciferase constructs and miR-18a mimics or mimics negativa control (mimics NC). The renilla constructs were also cotransfected as an internal control. Luciferase acticity was normalized to renilla luciferase activity. Data were presented as the mean ± SD of twice experiments with six replicates (Student’s t-test, *p < 0.05; **p < 0.01).

### Alkaline comet assay

The alkaline comet assay was performed according to the procedure of alkaline Comet assay kit (Genmed Scientifics, USA). The detailed protocol has been previously published [17]. Briefly, cells were pelleted and resuspended in 1 mL of PBS. Next, 10 μL of resuspended cells was mixed with an equal volume of prewarmed low melting- point agarose. The agarose-cell mixture was placed on fully frosted slide precoated with agarose and spread gently with a coverslip. After 10 min at 4 °C, the slides were immersed in precooled (4 °C) lysis solution for 80 min in a dark chamber. After soaking with electrophoresis buffer for 30 min, the slides were subjected to electrophoresis (25 V) for 30 min. Finally, the cells were stained with 10 μg/ml propidium iodide (Beyotime Biotechnology, ST511), and individual cells were viewed using an Olympus FSX100 fluorescence microscope.

### Array CGH

The detailed protocol has been previously published [17]. CGH was performed using Agilent SurePrint G3 Human Catalog 2x400K CGH Microarrays (Agilent, CA, USA). Genomic DNA of P3HR-1 or Raji cells transfected with miR-18a or mimics NC were extracted with the Universal Genomic DNA Kit (CW2298S, Cwbio, Beijing, China). The visualization and image analyses were performed using Feature Extraction (Agilent, Santa Clara, CA, USA).

### Statistical analysis

Survival data were analyzed by Kaplan-Meier analysis. The log-rank test was used to determine the difference among survival curves according to miR-18a and EBNA1. Pearson χ^2^-test was used to discover the association between miR-18a and EBNA1. The real-time PCR data were represented as the means ± standard deviation.Statistical significance was determined using a 2-tail Student’s t-test. Statistical analyses were performed by SPSS11.0 and GraphPad prism. For cell cycle analysis, statistical significance for fold over miRNA negative control was determined using a Student’s t-test. All *p* values were two-sided, and a p value of less than 0.05 was considered to be significant.

## Results

### Increased expression of miR-18a in lymphomas patients is associated with EBV infection and a shorter survival

**Fig. 1:**
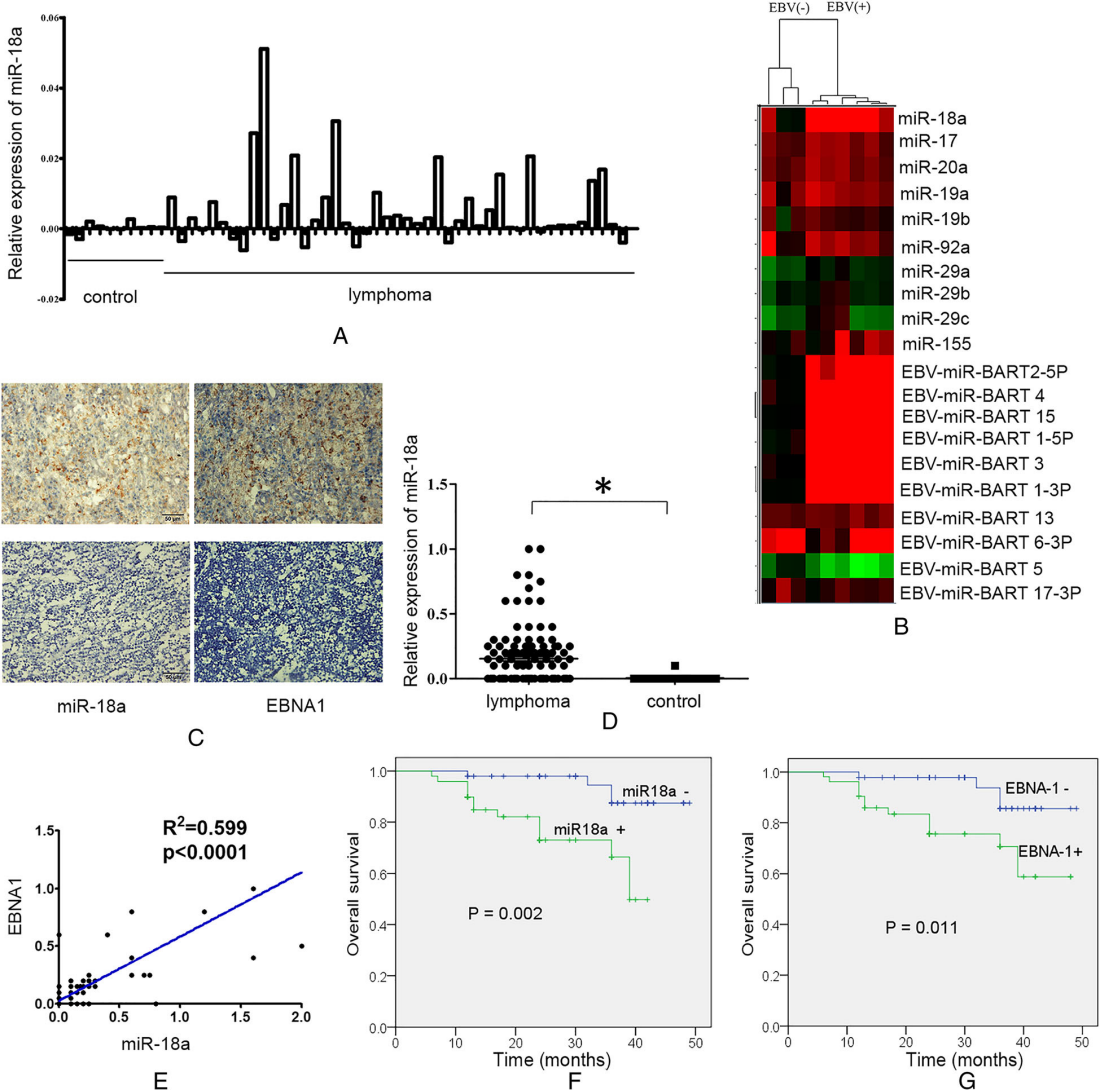
Expression of miR-18a in lymphoma patients. **a** Relative expression of miR-18a in diffuse large B-cell lymphoma patients and normal controls; publicly available microRNA array data (GSE42906) were compared between groups with GEO2R. **b** Unsupervised hierarchical clustering of microRNA expression. The miR-17-92 cluster and EBV-encoded microRNAs were differentially expressed between EBV- positive and -negative B cells; High and low expression levels are indicated by red and green, respectively. The raw data are shown in NCBI, GEO:GSE36926. **c** Expression of miR-18a and EBNA1. The expression of miR-18a was measured by in situ hybridization. The expression of EBNA1 was measured by immunohistochemistry. Representative figures are shown (× 100); Upper left and upper right: lymphoma biopsies; lower left and lower right: normal control lymph nodes. **d** Scatter plot of the observed expression scores of miR-18a. Expression was scored semi-quantitatively by multiplying the intensity and area of staining. **e** Correlation of the expression of EBNA1 and miR-18a. **f** Kaplan-Meier curves for patients according to the tumor expression of miR-18a. **g** Kaplan-Meier curves for patients according to the tumor expression of EBNA1

We first investigated the expressions of miR-18a and the miR-17-92 cluster in lymphomas samples and the association with EBV infection. Publicly available microRNA array data from 45 diffuse large B-cell lymphoma patients and 10 control lymph nodes or B cells with or without EBV infection were compared (GSE42906, GSE36926). Using GEO2R tool analysis, we found that the relative expression level of miR-18a was higher in B-cell lymphoma patients than in control lymph nodes (Fig. 1a). The unsupervised hierarchical clustering of microRNA expression showed that EBV-infected B cells had upregulated miR-17-92 cluster expression and were clustered together (Fig. 1b), indicating that the expression of the miR-17-92 cluster was correlated with the EBV infection status. miR-18a, which shares sequences with EBV-miRNA-BART5, was upregulated in EBV-infected B cells; however, EBV-miRNA-BART5 did not show upregulated expression in EBV-positive B cells. miR-155, which can be altered by EBV infection, was notably upregulated. miR-29a/b/c, which share sequences with EBV miRNA BART1-3p were downregulated.

The expressions levels of miR-18a and nuclear EBV protein EBNA1 in 100 lymphoid neoplasm tissues (59 of BL or DLBCL, 34 of NK/T-cell lymphomas and 7 of HL) and 20 non-cancerous control tissues were determined by in situ hybridization and immunohistochemistry. The expression levels of miR-18a and EBNA-1 across the entire cohort are described in Table 1. Compared with non-neoplastic lymphatic tissues, the expression of miR-18a in tumor biopsies from lymphomas was upregulated (*p* = 0.0127,Fig. 1c, d). Higher levels of miR-18a were correlated with advanced stage and extranodal lymph node metastasis (*p* < 0.01) but not with age and gender (Table 1). The expression of miR-18a was positively correlated with the expression of EBNA1 (Fig. 1e, Pearson r r: 0.7745, 95% confidence interval: 0.6813–0.8431, R squared: 0.5539, *P* < 0.0001). Higher expression levels of miR-18a and EBNA1 were correlated with a shorter overall survival (*p* = 0.002, *p* = 0.011, respectively, Fig. 1f, g).

### miR-18a promotes cell proliferation in EBV positive lymphoma cells and increases the EBV viral load

**Fig. 2:**
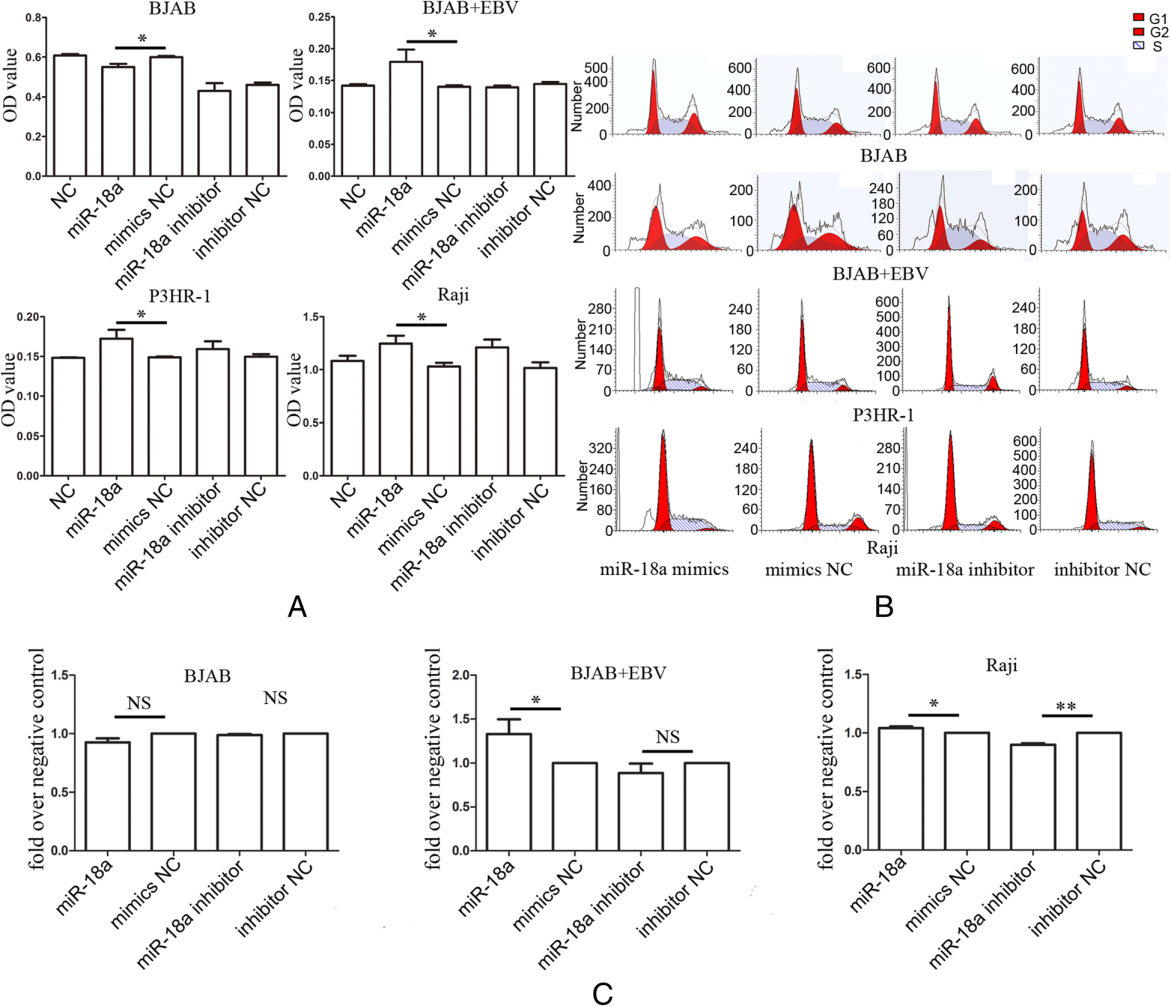
miR-18a promotes cell proliferation in EBV-positive lymphoma cells. **a** miR-18a promoted tumor cell growth in vitro in EBV-positive lymphoma cell lines. CCK-8 cell viability assay was performed after the transfection of miR-18a mimics or miR-18a inhibitor into EBV-positive lymphoma cell lines (P3HR-1, Raji, EBV infected BJAB) and the EBV-negative lymphoma cell line BJAB. The data represented the mean values of five repeats. The data are shown as the means±SD (Student T-test, *p < 0.05). **b** Flow cytometry analysis of the cell cycle after the transfection of miR-18a. **c** Cell cycle distribution of cells in S phase. The data were presented as the means±SD of four replicates (Student T-test, *p < 0.05; **p < 0.01)

We next investigated whether miR-18a affected lymphoma cell growth. Transfection of miR-18a into EBV-positive P3HR-1,Raji cells and EBV-infected BJAB cells resulted in promoted cell proliferation; however, when EBV-negative BJAB cells were transfected with miR-18a, decreased proliferation was observed (Fig. 2a), indicating that miR-18a promotes lymphoma cell growth in EBV-dependent manner. Cell cycle analysis indicated that miR-18a promoted the entry of G1 cells into S-phase in EBV-infected BJAB and EBV-positive Raji cells, miR-18a inhibitor caused a decrease in cell number in the S-phase of cell cycle in Raji cells, but in EBV-negative BJAB cells, the effect of miR-18a on cell cycle was not observed, indicating that the cell growth effects induced by miR-18a were associated with the status of EBV (Fig. 2b, c). In EBV-infected BJAB, miR-18a inhibitor did not show obvious effect on cell cycle, suggesting that miR-18a inhibitor may inhibit the reactivation of EBV, but does not reduce the burden of primary EBV infection (Fig. 2c).

**Fig. 3:**
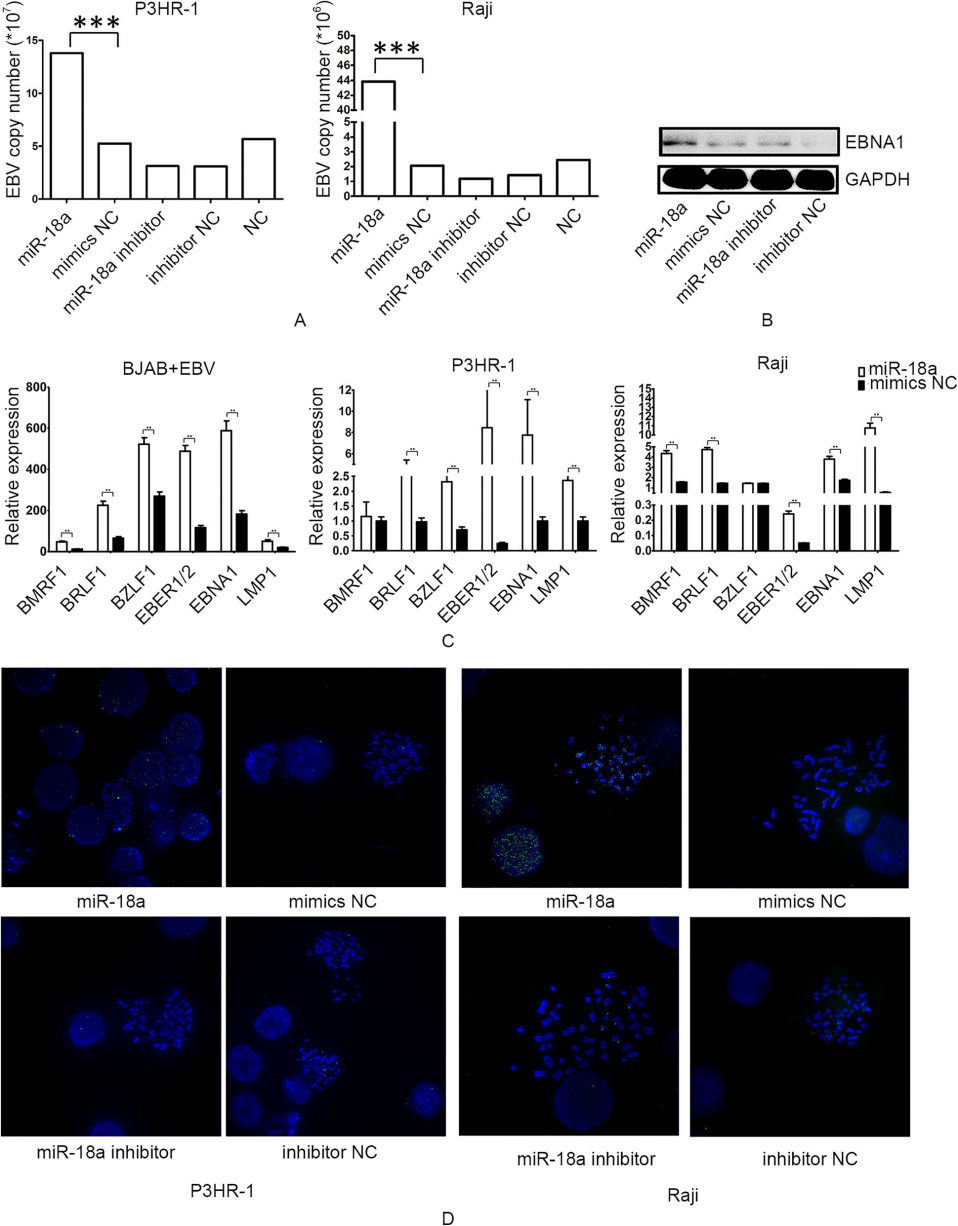
miR-18a increases the EBV viral load. **a** Transfection of miR-18a mimics in P3HR-1 and Raji resulted in a higher EBV viral load. Quantitative real-time EBV PCR was performed in samples collected from the culture media of cells. Viral DNA was extracted, and PCR was carried out according to the instructions. The EBV copy number was calculated according to a standard curve. **b** Expression of EBNA1 after transfection of miR-18a mimics and inhibitor in Raji cells as measured by western blotting. **c** Relative expression of EBV gene expression after transfection of miR-18a. **d** Visualization of episomal and integrated EBV DNA by fluorescence in situ hybridization

Transfection of miR-18a mimics in P3HR-1 and Raji cells resulted in a higher EBV viral load and EBV gene expression, including that of BZLF1, which controls entry into the EBV lytic replication phase (Fig. 3a-c). EBNA-1, which is always expressed in EBV-carrying proliferating cells, was overexpressed after the transfection of miR-18a (Fig. 3b). In situ hybridization generated more fluorescent spots in miR-18a-transfected P3HR-1, Raji and EBV-infected BJAB cells. EBV genomes staining reached the nucleus, being recognized as both latent punctate foci of episomal genomes and bright lytic staining (Fig. 3d, Additional file 1: Figure S1). There were no positively stained spots in EBV-negative BJAB (Additional file 1: Figure S1).

### miR-18a is a DNA damage sensor and mediates EBV reactivation after DNA damage

**Fig. 4:**
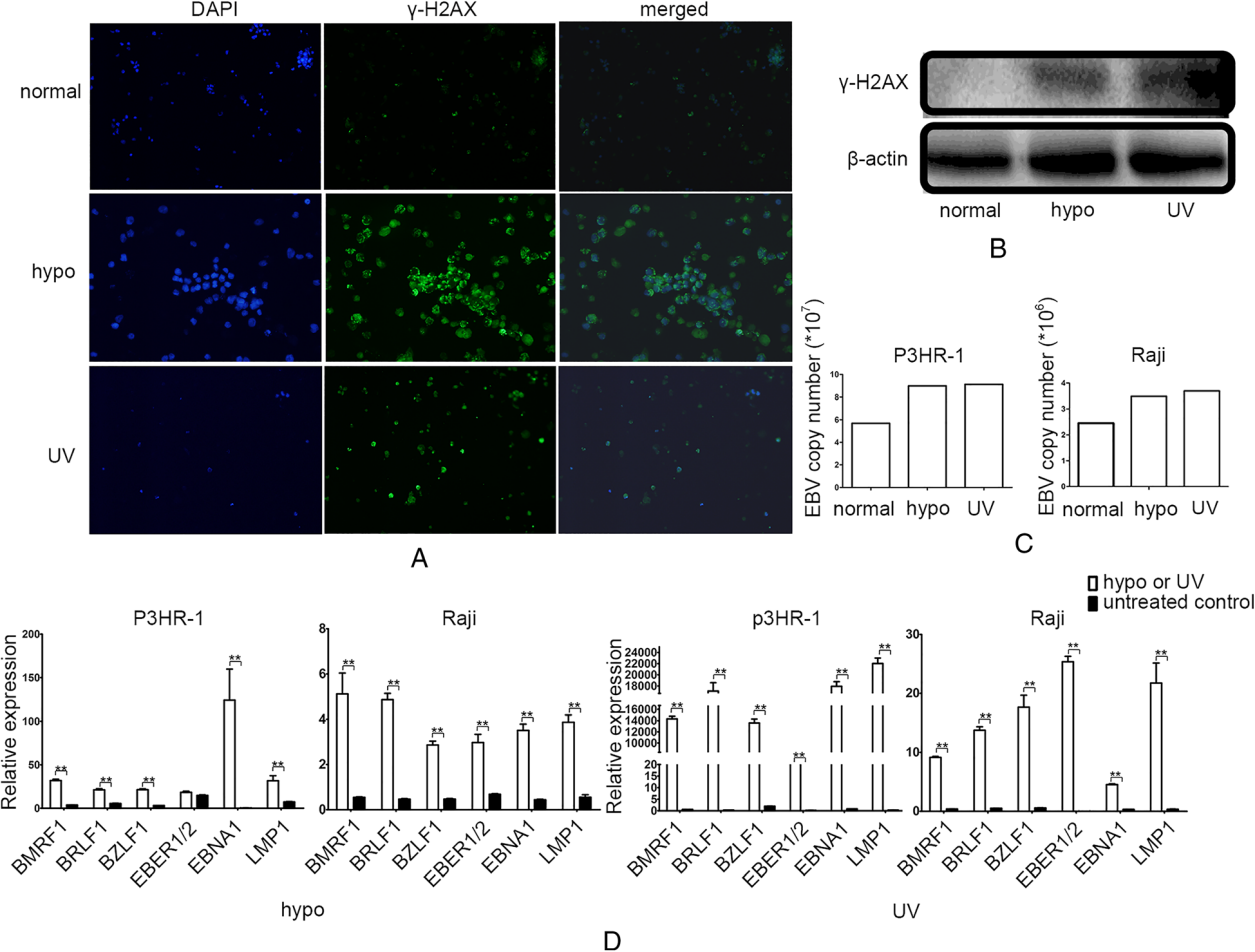
DNA damage reactivates EBV. **a** Expression of γ-H2AX as measured by immunofluorescence after treatment with hypoxia or UV. EBV-positive Raji cells were stained with anti-γ-H2AX and DAPI. Expression of γ-H2AX is indicated as green loci. DAPI was used to stain the cell nuclei. The merge images present the DAPI and FITC as blue and green, respectively. **b** Expression of γ-H2AX as measured by western blotting after treatment with hypoxia or UV in Raji cells. β-actin served as an loading control. **c** EBV copy number as measured by real-time PCR after treatment with hypoxia or UV. **d** Relative expression of EBV genes as measured by real-time PCR after treatment with hypoxia or UV

Studies have revealed that DNA-damaging agents, including chemotherapy,hypoxia and radiation, can reactivate EBV [11, 18, 19]. The expression of γ-H2AX, which is the biomarker for DNA damage, was increased in EBV- positive Raji cells after treatment with UV or hypoxia (Fig. 4a, b). We observed that UV exposure and hypoxia treatment both caused an increase in the EBV viral load in EBV-positive lymphoma cells (Fig. 4c). The EBV-related gene expression was dramatically increased after treatment with UV or hypoxia, suggesting that hyperproliferated EBV was mediated by the DNA damage response induced by UV or hypoxia (Fig. 4d).

**Fig. 5:**
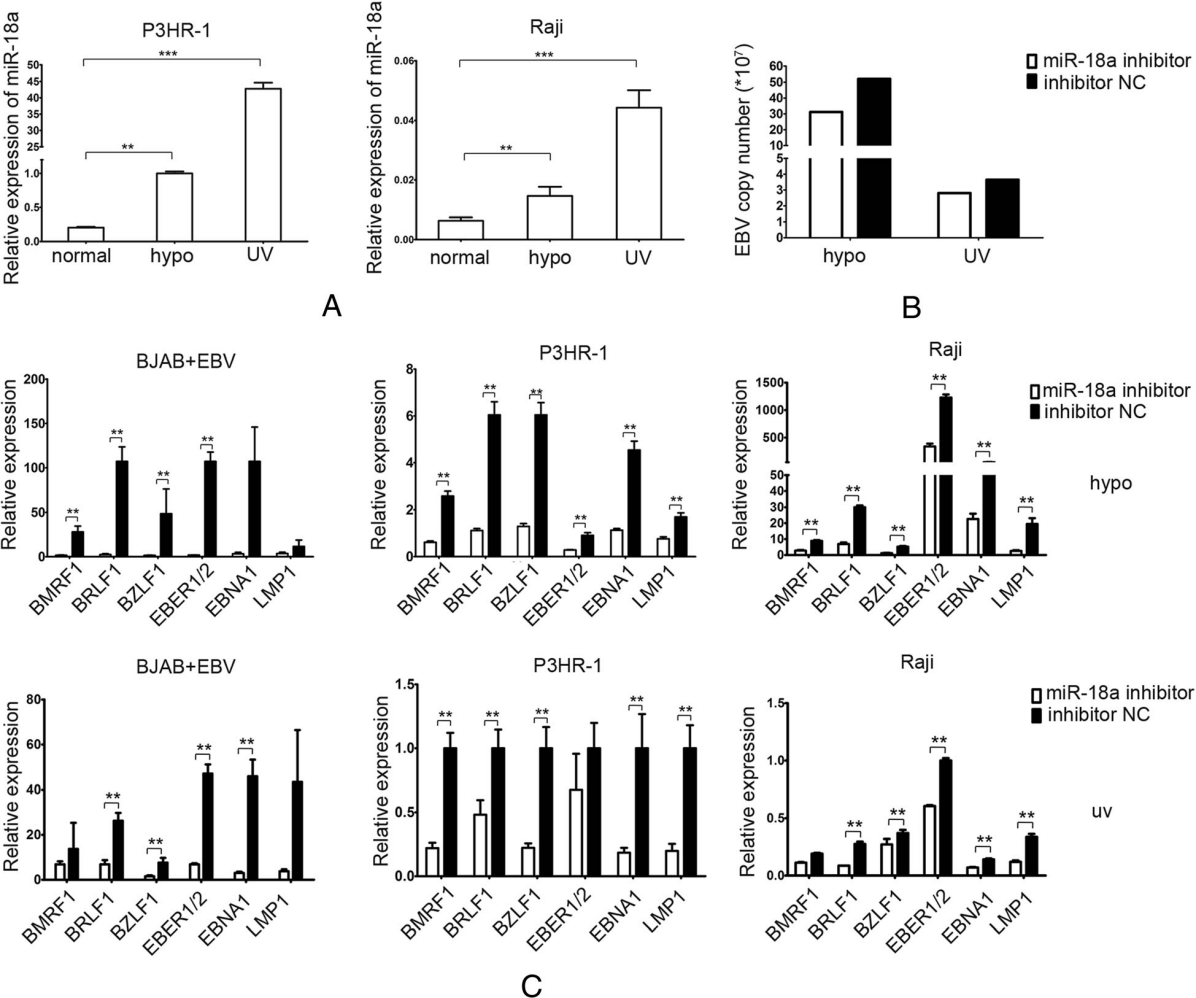
DNA-damaging agents reactivate EBV through miR-18a. **a** Relative expression of miR-18a after treatment with hypoxia or UV. Real-time PCR was used to measure the mRNA expression of EBV-related genes. β-actin served as an internal control. **b** miR-18a inhibitor decreased the EBV copy number induced by hypoxia or UV in Raji cells. **c** miR-18a inhibitor decreased EBV gene expression induced by hypoxia or UV. Relative expression of EBV genes as measured by real-time PCR. upper: hypoxia treatment; lower: UV treatment

To determine whether DNA-damaging agents reactivate the EBV genome through miR-18a, we measured the expression of miR-18a upon treatment with UV and hypoxia. After UV exposure and hypoxic treatment, Raji and P3HR-1 showed a significant increase in miR-18a, suggesting that miR-18a is a DNA damage sensor (Fig. 5a). We also found that EBV infection can increase the expression of miR-18a, which may occur because of the DNA damage arising from EBV infection (Additional file 1: Figure S2). Inhibition of miR-18a can reduce the EBV copy and the EBV-related gene expression in EBV-positive lymphoma cell line Raji after exposure to hypoxia and UV (Fig. 5b, c), indicating that UV exposure or hypoxic treatment caused EBV reactivation through miR-18a expression.

### miR-18a reactivated EBV by targeting ATM-mediated DDR

**Fig. 6:**
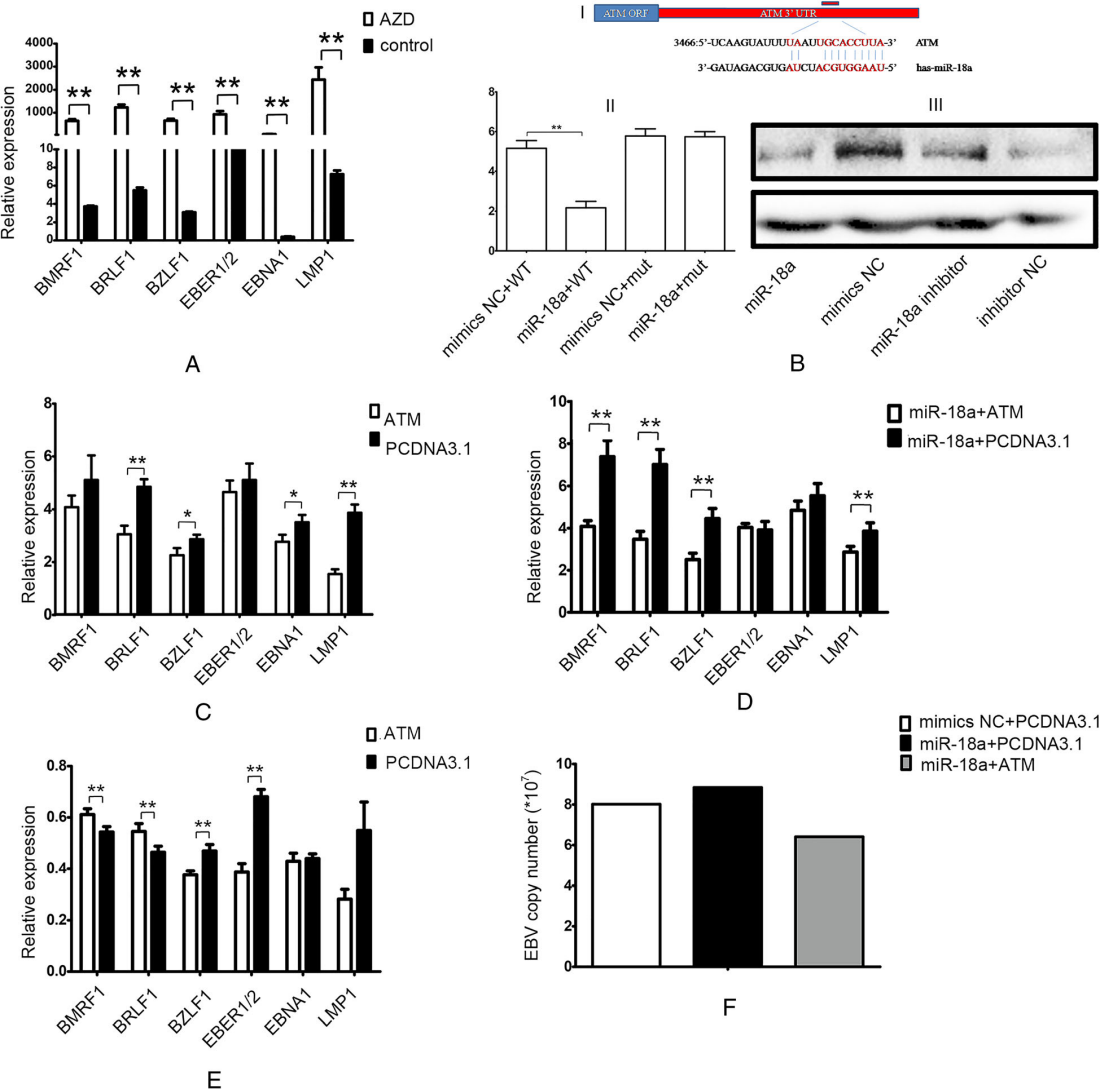
miR-18a reactivates EBV by targeting ATM. **a** DNA-PK inhibitor AZD 8055 increased EBV gene expression in Raji cells. Real-time PCR was used to measure the mRNA expression of EBV-related genes. GAPDH served as an internal control. **b** ATM is a potential target of miR-18a. I: Schematic representation of the 3’UTR of ATM. The red bar shows the predicted miR-18a binding sites in the 3’UTR of ATM. The sequence of mature miR-18a aligned to target sites is shown. (II) Luciferase activity assay. The reporter constructs in which the 3’UTR of ATM, wild-type or miR-18a binding-site mutant, was cloned downstream of the luciferase open reading frame. 293 cells were co-transfected with the luciferase construct and miR-18a mimics or control miRNA. The renilla construct was also cotransfected as an internal control. Luciferase activity was normalized to renilla luciferase activity. The data were presented as the means±SD of two experiments with six replicates (Student T-test, *p < 0.05; **p < 0.01; ***p < 0.001). III: miR-18a decreased the expression of ATM. Western blot of ATM was performed 48 h after the transfection of miR-18a mimics and miR-18a inhibitor. GAPDH was used as an internal control. **c** ATM inhibited the expression of EBV-related genes under hypoxic conditions (*p < 0.05; **p < 0.01). **d** ATM reversed the promotion effect of miR-18a on the EBV-related gene expression (*p < 0.05; **p < 0.01). **e** EBV gene expression after transfection of ATM under normoxia (*p < 0.05; **p < 0.01). **f** Transfection with ATM inhibited the promotion effect of miR-18a on the EBV viral load

The components of the DNA damage signalling pathway, including ATM, ATR and DNA-PK, play key roles in defending against neoplastic transformation. The DNA-PK inhibitor AZD 8055 can increase EBV-related gene expression (Fig. 6a). As reactivation of EBV can occur with DNA damage caused by UV exposure or hypoxic treatment through miR-18a, we further investigated the roles of miR-18a in DNA damage. Previous research has shown that ATM is a potential target of miR-18a [20]. The schematic diagram of miR-18a binding sites in the 3’UTR of ATM is shown in Fig. 6b. Transfection with miR-18a mimics led to a significant decrease in luciferase activity compared with the miRNA control. By contrast, luciferase activities of mutant 3’ UTR remained unchanged in miR-18a-overexpressing cells (Fig. 6b). Western blot analysis showed that the over-expression of miR-18a suppressed endogenous ATM expression in EBV-infected BJAB cells (Fig. 6b). However, the inhibitory effect of miR-18a on ATM was not obvious in EBV-negative BJAB cells (Additional file 1: Figure S3).

Under hypoxic conditions, transfection with ATM could inhibit the expression of EBV-related genes and reverse the promotion effect of miR-18a on the EBV-related gene expression (Fig. 6c, d). Conversely, under normoxia, transfection with ATM increased EBV lytic gene expression (BMRF1,BRLF1) and decreased the expression of EBV lytic gene BZLF1 and latent genes (EBER1/2, LMP1) (Fig. 6e). Transfection with ATM inhibited the promotion effect of miR-18a on the EBV viral load (Fig. 6f). These results indicated that miR-18a reactivated EBV through inhibiting ATM expression.

### miR-18a promotes genomic instability through the reactivation of EBV

**Fig. 7:**
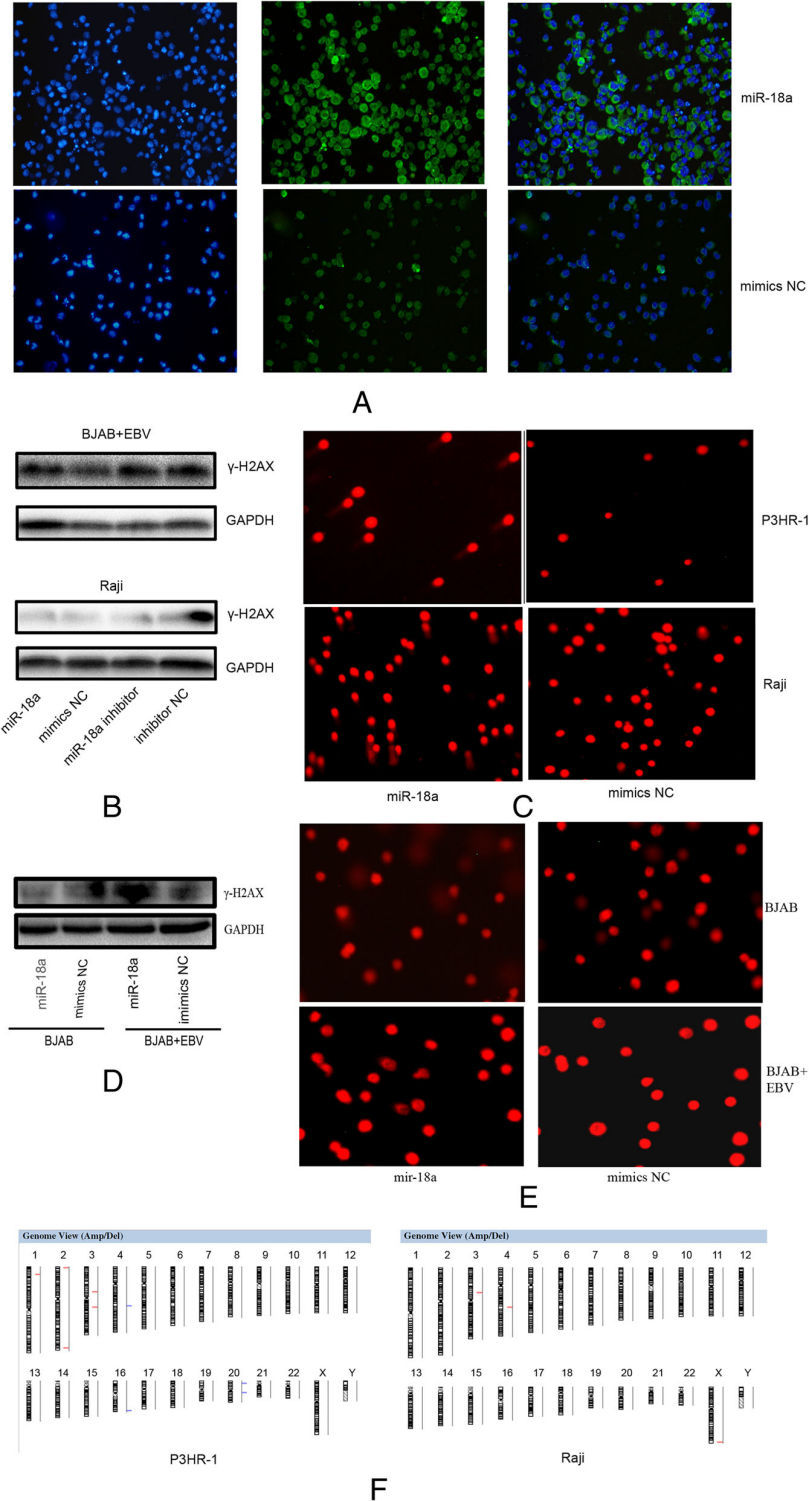
miR-18a induces DNA damage. **a** Expression of γ-H2AX as measured by immunofluorescence in Raji cells; EBV-positive Raji cells were stained with anti-γ-H2AX and DAPI. Expression of γ-H2AX is indicated as green loci. DAPI was used to stain the cell nuclei. The merge images present the DAPI and FITC as blue and green, respectively. **b** Expression of γ-H2AX as measured by western blotting in EBV-positive or -negative cells. **c** Detection of DNA damage after transfection of miR-18a. The comet assay was applied. Cells were electrophoresed in agarose gels on a coverslip and were stained with propidium iodide. Labeled DNA was visualized under a fluorescence microscope. **d** Detection of DNA damage after transfection of miR-18a in EBV-negative and -positive BJAB cells. Magnification, × 100. **e** Graphic presentation of all chromosomal changes. Cells transfected with miR-18a and mimics negative control were analyzed by comparative genomic hybridization array (Array-CGH). The regions of DNA gain (blue) and loss (red) are shown

Because miR-18a targets ATM and impairs the DNA damage response, we further investigated whether miR-18a affected genomic stability. When transfected with miR-18a,EBV-positive Raji cells showed increased expression of γ-H2AX (Fig. 7a, b). The Comet assay showed long comet tails after transfection of miR-18a in P3HR-1 and Raji cells (Fig. 7c, d). However, transfection of miR-18a in EBV-negative BJAB cells did not show obvious overexpression of γ-H2AX and long comet tails, indicating that miR-18a caused DNA damage through EBV reactivation (Fig. 7b, d).

DNA damage that is not repaired leads to genomic instability and cancer. In an Array-CGH assay, the evaluation of DNA copy number changes was performed by comparing a DNA test isolated from Raji cells and P3HR-1 cells, which were transfected with miR-18a against a normal reference DNA of control lymphoma cells. A graphical presentation of the regions of gain (blue) and loss (red) is shown in Fig. 7e. These abnormalities in cells when transfected with miR-18a included gains in chromosome 16 and 20. Losses in chromosome 1, 2, 3 and chromosome X are also shown (Fig. 7e). Thus, miR-18a transfection inhibited ATM-mediated DNA repair and reactivated EBV, leading to genomic instability.

## Discussion

In this study, we performed a comprehensive analysis of expression of the miR-17-92 cluster and EBV status regarding lymphomas. Overexpression of miR-18a is observed in lymphomas tissue and EBV-infected B cells. We demonstrated that miR-18a can reactivate EBV through inhibiting the DNA damage response and induce genomic instability through EBV reactivation.

EBV established a persistent, asymptomatic, latent infection in a restricted pool of resting B cells [6, 21]. In immunocompetent adults, the EBV viral load is stable for years. It is assumed that the latently infected memory B cells undergo periodic reactivation to produce infectious virus [22]. EBV reactivation begins through expression of the immediate-early transcription factors BZLF1, which initiates a cascade of expression of early genes and late genes [23]. The roles of EBV in the lytic or latent state in the development of lymphoma are not fully understood. B cell transformation after EBV infection in vitro is believed to be a latent infection. However, the in vitro model is not consistent with the viral gene expression programs observed in most EBV-positive B-cell tumors [24]. Although the EBV latent gene-encoded products activate survival pathways to promote the progression of cancer and cellular transformation, an increased level of lytically infected cells may increase the likelihood of EBV-associated malignancies [25, 26]. EBV viral load measurement is suggested to predict and monitor EBV-associated tumors, including nasopharyngeal carcinoma, post-transplant lymphoproliferative disorder, and Hodgkin’s disease [27]. AIDS patients and organ transplant patients, who have a high risk of developing EBV-associated lymphomas, also have a high level of lytic as well as latent EBV infection [26, 28]. A high level of EBV particles would be predicted to increase the number of latently infected B cells. The differentiation of EBV-infected memory B cells into plasma cells and acute stress represent two distinct pathways of EBV reactivation [23]. Although this treatment is controversial, antiviral drugs that block virus replication and kill proliferating infected cells were administered to EBV-positive lymphoma patients [29]. Our data showed that miR-18a caused a burst of lytic gene expression and concomitant transcription of EBV latent gene expression. miR-18a promoted the proliferation of EBV-positive lymphoma cells but not EBV-negative lymphoma cells, suggesting that miR-18a-induced EBV reactivation mediated the increase in lymphoma cell growth.

Although EBV infection is related to B cell malignancy, the slow course of cancer induction and infections worldwide suggest that EBV alone is rarely sufficient to cause lymphoma. EBV infection, endogenous and exogenous damages in DNA and host repair mechanisms play synergistic effects on tumorigenesis of lymphomas. Exposure to DNA damaging agents, such as certain chemotherapy, ultraviolet or hypoxia, can induce the reactivation of viral replication, causing fold increases in the EBV viral load [6, 21]. The replication of EBV, in turn, increases replicative stress, similar to oncogene activation, leading to the induction of the DNA damage response (DDR). DDR emerges as a barrier to tumor progression responding to cellular DNA replicative stress [30]. The host DDR, which is orchestrated by ATM and ATR kinase, senses EBV-induced oncogenic stress,causes cell cycle arrest or apoptosis and blocks long-term outgrowth of most infected cells [19] [31]. Inhibition of the DDR kinase ATM markedly increases the transformation efficiency of primary B cells [19]. ATM kinase expression is reduced in EBV-associated nasopharyngeal carcinomas [32]. It was reported that the DNA repair sensor ATM causes EBV lytic reactivation in Burkitt lymphoma cells in p53-dependent and non-dependent ways [19]. The EBV latent-to-lytic switch is mediated by the viral proteins BZLF1, BRLF1 and BRRF1 [11]. The ability of the EBV BRLF1 and BRRF1 proteins to induce lytic reactivation in EBV-infected AGS cells is ATM dependent. However, overexpression of BZLF1 induces lytic gene expression in the presence or absence of ATM activity [19]. ATM enhances BZLF1 promoter activity in the context of the intact EBV genome [11]. In our study, under normoxia, ATM induced the overexpression of BRLF1 and BMRF1, however, decreased the expression of BZLF1 in EBV-positive Raji cells. Under DNA damage stress, ATM decreased lytic and latent EBV gene expressions and reversed the induction of miR-18a on the EBV viral load. We speculated that ATM decreased EBV gene expression, including lytic or latent genes in the context of EBV- or DNA damage agents-induced genomic stress. As a host mechanism, miR-18a acts as a sensor to DNA damage stress induced by hypoxia, UV and EBV infection, inhibiting DDR and promoting genomic instability. miR-18a inhibited ATM-mediated DDR and increased the virus proliferation, increasing the growth of lymphomas and indicating the oncogenic effect of EBV is dependent on the host microenvironment. DNA damage that is not repaired leads to genomic instability and cancer. It is now generally accepted that oncoproteins encoded by tumor viruses can drive genomic instability and initiate tumorigenesis. EBV infection promotes genomic instability and telomere dysfunction [12]. Repeated malarial infection helps EBV to cause lymphomas. However, it was reported that antibodies to fight malaria cause DNA damage that can lead to Burkitt’s lymphoma because of the ligation of inappropriate segments of chromosomes during antibody affinity maturation [33]. Thus, host factors contribute to the genomic instability induced by viral replication.

There are three EBV-encoded microRNAs that share sequences with human miRNAs: EBV miRNA BART1-3p with human miRNA miR-29a/b/c; BART5-5p with miR-18a/b and BART22-3p with miR-520d-5p, and miR-524-5p [14, 34]. EBV-encoded microRNAs have been reported to regulate human and viral transcripts. EBV can also alter host miRNA expression [34]. BART miRNAs are present in all EBV-infected cells, with much higher expression in epithelial cells than in infected B lymphocytes. BART miRNAs act as repressors of EBV lytic replication and probably maintain a balance between the virus and its host [35]. In our study, EBV can enhance the expression of miR-18a (Additional file 1: Figure S3). In contrast to the roles of BART miRNAs, the host miR-18a promoted EBV replication. Most EBV miRNAs co-target mRNAs with host miRNAs, in particular with members of the miR-17-92 miRNA cluster [34]. Our study found that ATM is targeted by miR-18a in EBV-infected BJAB cells but not in EBV negative BJAB, indicating that EBV miRNAs co-target mRNAs with host miRNAs. Although sharing seed sequence identity, miR-18a and BART5-5p showed different potential to regulate expression of LMP1. BART5-5p can repress the expression of LMP1 and not miR-18a-5p [34].

## Conclusions

Taken together, this study’s results demonstrate the interplay of host factors, environmental factors and EBV infection status. Environmental genotoxic stresses, such as UV and we found the interplay of host factors, environmental factors and EBV infection status. Environmental genotoxic stresses, such as UV and hypoxia, caused EBV reactivation and overexpression of miR-18a. miR-18a targeted ATM and inhibited host DDR, thereby causing EBV reactivation and genomic instability and contributing to the development of lymphoproliferative diseases. Inhibition of miR-18a may be a novel approach to prevent the reactivation of EBV.

## Additional file

Additional file 1: **Figure S1.** Visualization of episomal and integrated EBV DNA by fluorescence in situ hybridization in EBV-positive or -negative BJAB cells. **Figure S2.** EBV infection increased the expression of miR-18a. Real-time PCR was used to measure the mRNA expression of EBV-related genes. **Figure S3.** Expression of ATM as measured by western blotting after transfection of miR-18a in EBV-negative BJAB. cells. (ZIP 3643 kb)

## Abbreviations

3′UTR: 3′-untraslated region
BL: Burkitt lymphoma
DDR: DNA damage response
DLBCL: Diffuse large B-cell lymphoma
EBV: Epstein-Barr virus
HL: Hodgkin lymphomas
IM: Infectious mononucleosis
LCL: Immortalized lymphoblastoid cell lines
miRNA: Micro RNA
NC: Negative control
NHLs: Non-Hodgkin lymphomas
NPC: Nasopharyngeal carcinoma
qRT-PCR: Quantitative real-time polymerase chain reaction
