# Correction to: miR-18a reactivates the Epstein-Barr virus through defective DNA damage response and promotes genomic instability in EBV-associated lymphomas

**DOI:** 10.1186/s12885-019-5378-x

**Published:** 2019-03-01

**Authors:** Pengfei Cao, Meili Zhang, Lujuan Wang, Buqing Sai, Jiuqi Tang, Zhaohui Luo, Cijun Shuai, Liyang Zhang, Zheng Li, Yanjin Wang, Guiyuan Li, Juanjuan Xiang

**Affiliations:** 1 Key Laboratory of Carcinogenesis of Ministry of Health, Xiangya Hospital, Central South University, Changsha, 410078 Hunan China; 2 Key Laboratory of Carcinogenesis and Cancer Invasion of Ministry of EducationCancer Research Institute, Central South University, Changsha, 410078 Hunan China; 3 Hunan Key Laboratory of Nonresolving inflammation and Cancer, Desease Genome Research Center, The Third Xiangya Hospital, Central South University, Changsha, 410013 Hunan China; 4 State Key Laboratory of High Performance Complex Manufacturing, Central South University, Changsha, 410083 Hunan China; 5 People’s Hospital of Dezhou, Dezhou, 253045 Shandong China; 6 Department of Neurosurgery, Xiangya Hospital, Central South University, Changsha, 410078 Hunan China

Following publication of the original article [1], the authors reported that they had supplied the incorrect figure for publication.

**Cao et al. BMC Cancer (2018) 18:1293.**

**https://doi.org/10.1186/s12885-018-5205-9**

Following publication of the original article [1], the authors reported that they had supplied the incorrect figure for publication. The correct figure is displayed below. The authors apologise for the error.

**Fig. 2:**
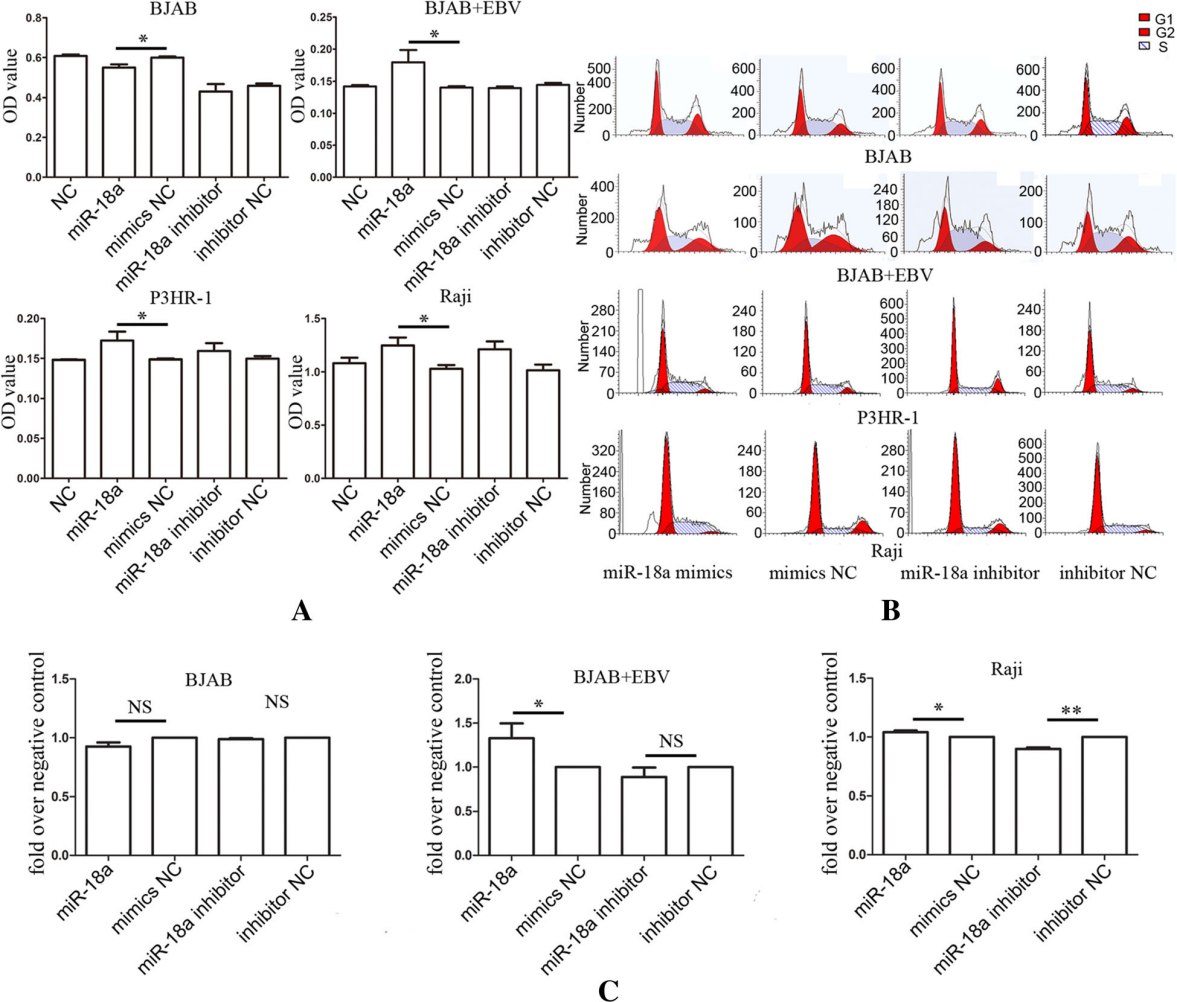
miR-18a promotes cell proliferation in EBV-positive lymphoma cells. **a** miR-18a promoted tumor cell growth in vitro in EBV-positive lymphoma cell lines. CCK-8 cell viability assay was performed after the transfection of miR-18a mimics or miR-18a inhibitor into EBV-positive lymphoma cell lines (P3HR-1, Raji, EBV infected BJAB) and the EBV-negative lymphoma cell line BJAB. The data represented the mean values of five repeats. The data are shown as the means±SD (Student T-test, *p < 0.05). **b** Flow cytometry analysis of the cell cycle after the transfection of miR-18a. **c** Cell cycle distribution of cells in S phase. The data were presented as the means±SD of four replicates (Student T-test, *p < 0.05; **p < 0.01)
