# Supplementary figures and images for: miR-18a reactivates the Epstein-Barr virus through defective DNA damage response and promotes genomic instability in EBV-associated lymphomas
Source: BMC Cancer. 2018 Dec 29;18:1293. doi: 10.1186/s12885-018-5205-9 (PMC6311029; doi:10.1186/s12885-018-5205-9)

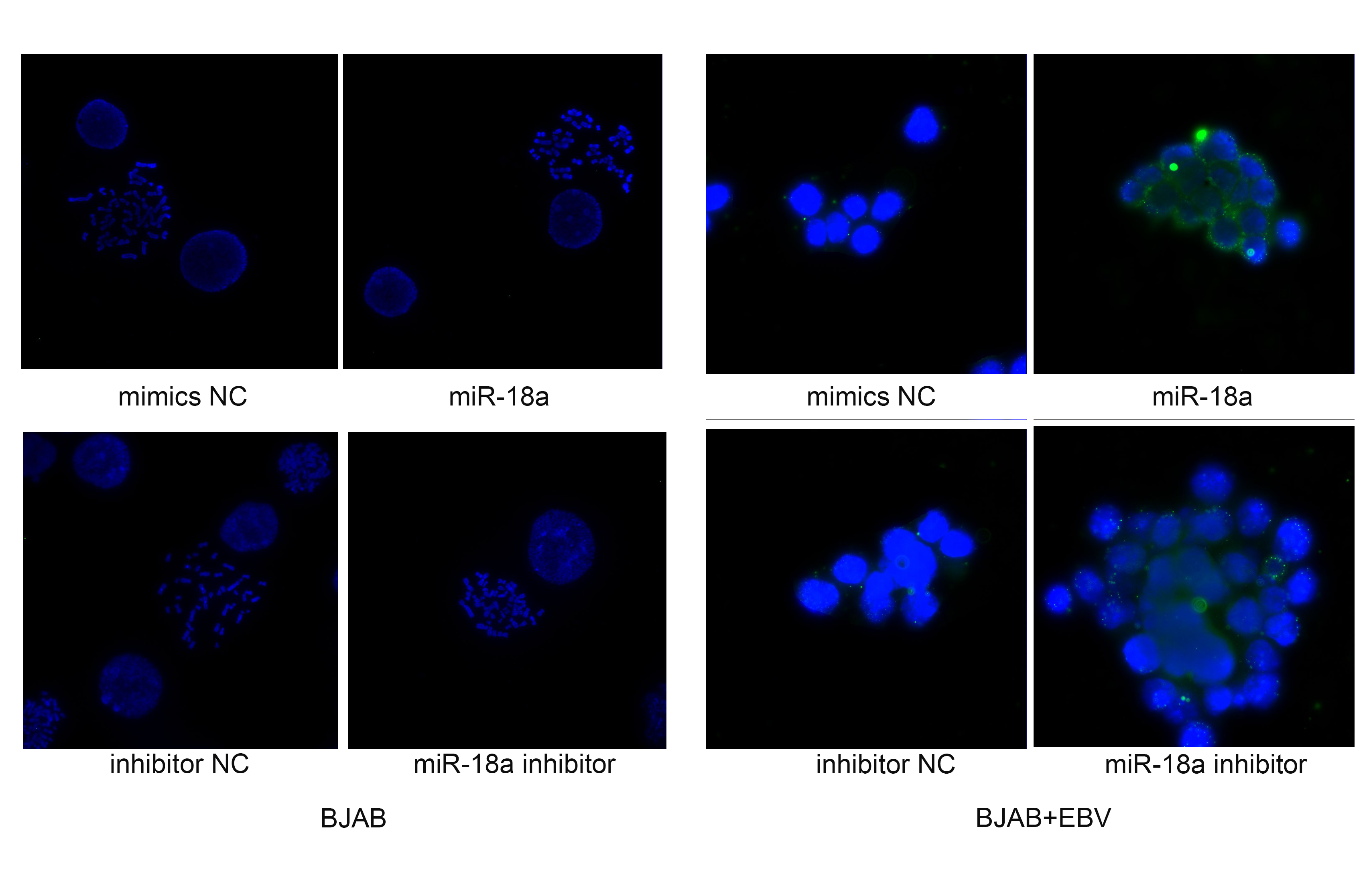

Supplement: Supplementary file 1 — Figure S1. Visualization of episomal and integrated EBV DNA by fluorescence in situ hybridization in EBV-positive or -negative BJAB cells. Figure S2. EBV infection increased the expression of miR-18a. Real-time PCR was used to measure the mRNA expression of EBV-related genes. Figure S3. Expression of ATM as measured by western blotting after transfection of miR-18a in EBV-negative BJAB. cells. (ZIP 3643 kb) [file 12885_2018_5205_MOESM1_ESM.zip › supplementary figure 1R4.tif]

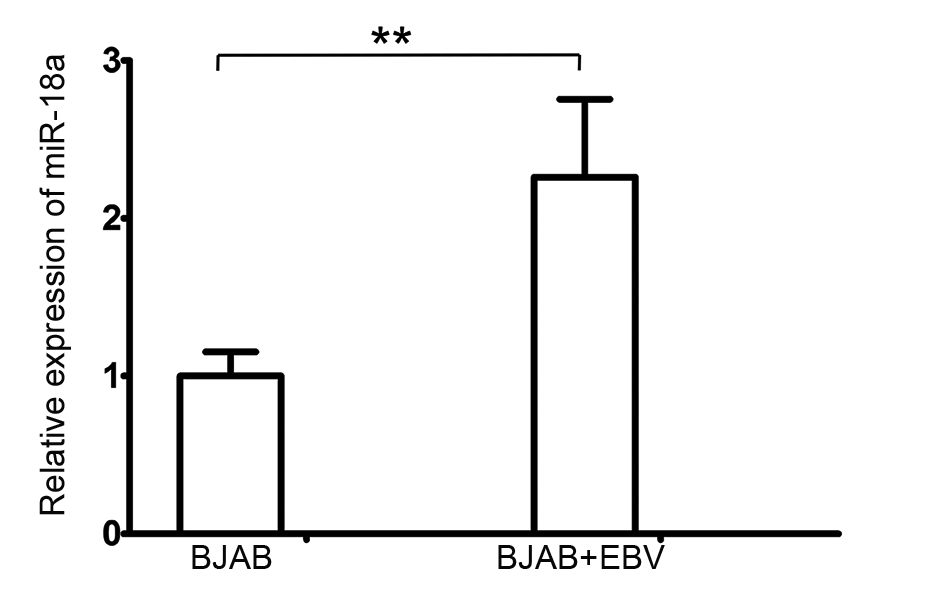

Supplement: Supplementary file 1 — Figure S1. Visualization of episomal and integrated EBV DNA by fluorescence in situ hybridization in EBV-positive or -negative BJAB cells. Figure S2. EBV infection increased the expression of miR-18a. Real-time PCR was used to measure the mRNA expression of EBV-related genes. Figure S3. Expression of ATM as measured by western blotting after transfection of miR-18a in EBV-negative BJAB. cells. (ZIP 3643 kb) [file 12885_2018_5205_MOESM1_ESM.zip › supplementary figure 2R4.tif]

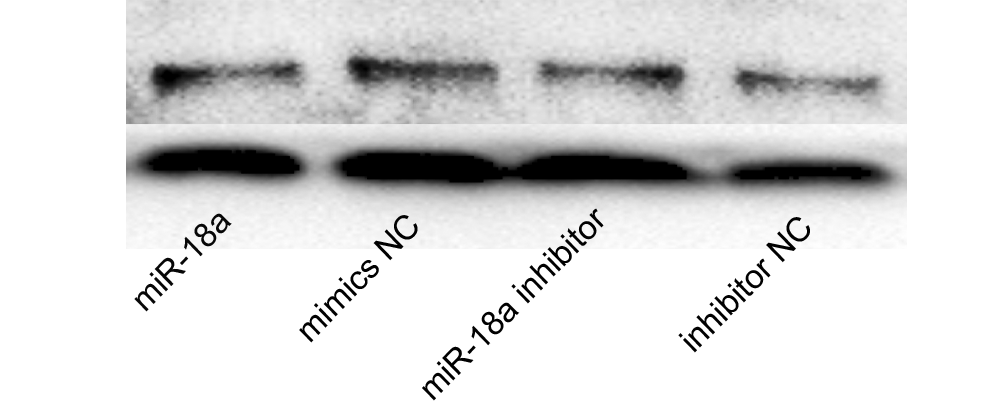

Supplement: Supplementary file 1 — Figure S1. Visualization of episomal and integrated EBV DNA by fluorescence in situ hybridization in EBV-positive or -negative BJAB cells. Figure S2. EBV infection increased the expression of miR-18a. Real-time PCR was used to measure the mRNA expression of EBV-related genes. Figure S3. Expression of ATM as measured by western blotting after transfection of miR-18a in EBV-negative BJAB. cells. (ZIP 3643 kb) [file 12885_2018_5205_MOESM1_ESM.zip › supplementary figure 3R4.tif]
